# Acute-on-Chronic Liver Failure From Acquired Hemochromatosis in a Patient With Sickle Cell Disease

**DOI:** 10.7759/cureus.46959

**Published:** 2023-10-13

**Authors:** Matthew B Anderson, Ryan Layton, Ryan Woods

**Affiliations:** 1 Internal Medicine, Atrium Health Wake Forest Baptist, Winston Salem, USA; 2 Internal Medicine, Wake Forest School of Medicine, Winston Salem, USA; 3 Hematology and Oncology, Atrium Health Wake Forest Baptist, Winston Salem, USA

**Keywords:** acute-on-chronic liver failure (aclf), aclf, end stage renal disease (esrd), extreme hyperbilirubinemia, acute-on-chronic liver failure, sickle cell intrahepatic cholestasis, sickle cell disease (scd)

## Abstract

Late presentations of liver failure were previously rare in clinical practice given the high mortality of sickle cell disease (SCD) and shorter life expectancy compared to the general population. With advancements in therapeutics for SCD, patients with SCD have increased lifespans, and with them, patients are placed at increased risk for differing patterns of chronic and end-organ failures. We describe a case of an elderly patient who had multiple chronic complications from her years of SCD, including end-stage renal disease (ESRD) on hemodialysis, acquired hemochromatosis, cirrhosis, and pulmonary hypertension. During this presentation for shortness of breath, she developed acute-on-chronic liver failure (ACLF) with a significant lower gastrointestinal bleed and hemorrhagic shock. Her family ultimately elected to pursue comfort care measures, and she passed later that day.

## Introduction

Acute-on-chronic liver failure (ACLF) is a systemic inflammatory state associated with organ failure that is associated with high short-term mortality [1]. As the population of patients diagnosed with sickle cell disease (SCD) lives longer, particularly in resource-rich settings, more late-term complications from multi-organ failures will be prevalent [2]. Patients dependent on red blood cell transfusions, iron overload, and chronic liver disease place these patients at risk for hepatic and other complications [3]. We present the case of an elderly patient with SCD who later developed ACLF at the end stage of her SCD, and through this case, we aim to increase awareness of such complications and the importance of additional research for treatment strategies.

This case was previously presented as a poster presentation at the 2023 Wake Forest University School of Medicine Department of Internal Medicine Research Symposium on May 10, 2023 [4].

## Case presentation

**Figure 1:**
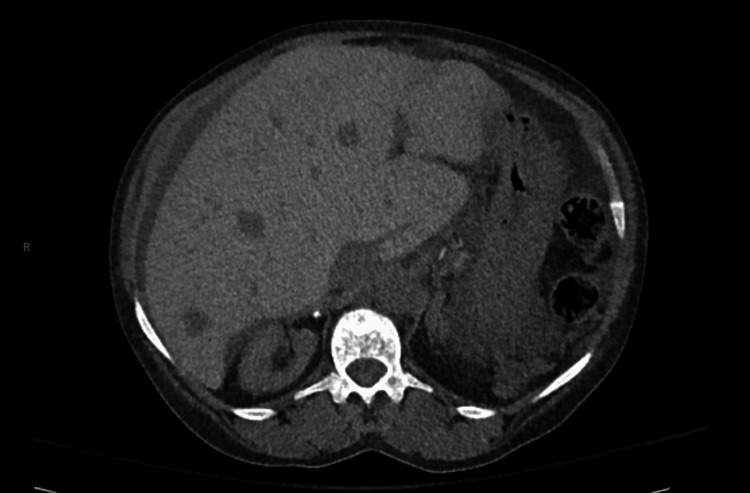
Abdominal CT, axial view, demonstrating hepatomegaly

**Figure 2:**
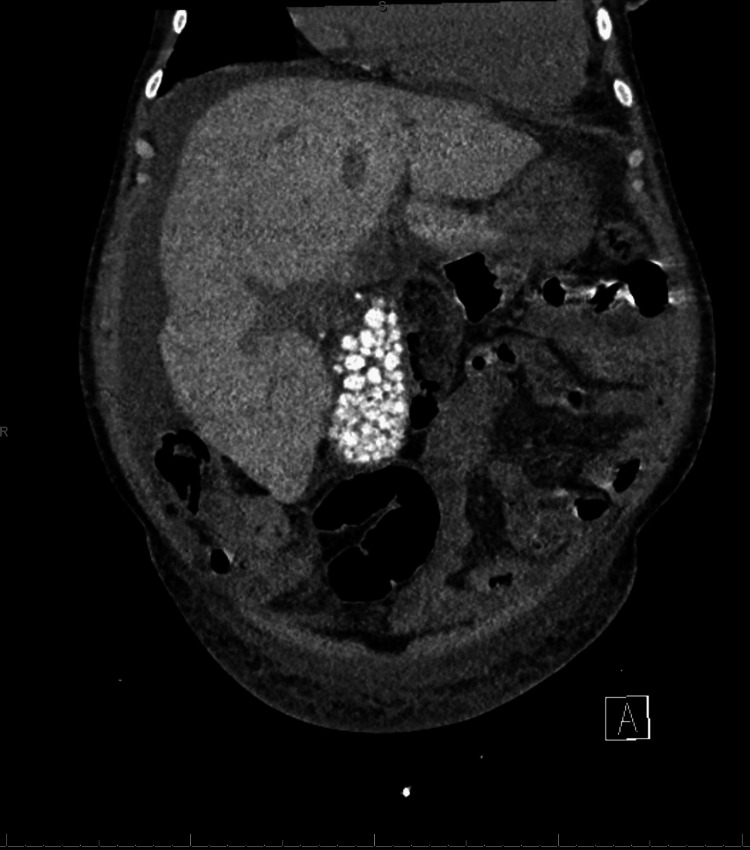
Abdominal CT, coronal view, demonstrating hepatomegaly and cholelithiasis

A 63-year-old female with a history of SCD, end-stage renal disease (ESRD) on hemodialysis, pulmonary hypertension, and acquired hemochromatosis from transfusion dependence presented to the emergency department after two weeks of increased shortness of breath and fatigue. She was intermittently taking deferasirox for her chronic iron overload due to GI intolerance and relative contraindications in ESRD. She continued to receive transfusions every other week for her chronic anemia and hemodialysis four times a week. On presentation, she denied any bleeding, melena, hematochezia, or hematemesis. Vital signs on arrival included a blood pressure of 125/46, a heart rate of 49 beats per minute, a respiratory rate of 18 breaths per minute, a temperature of 98.4F, and a SpO_2_ of 95% on room air. On the physical exam, she was noted to have scleral icterus and jaundice with non-tender hepatomegaly on the abdominal exam. Initial lab findings included a hemoglobin of 7.8 g/dL (reference range for women: 12.3-15.3 g/dL), 10.9% reticulocytes (reference range for adults: 0.5-2.2%), white blood cell (WBC) count of 19,700/uL (reference range: 4400-11,000/uL), platelets of 228,000/uL (reference range: 150,000-450,000/uL), serum creatinine of 6.39 mg/dL (reference range: 0.5-1.5 mg/dL), serum total bilirubin of 52.9 mg/dL (reference range: 0.1-1.2 mg/dL), direct bilirubin of 36.7 mg/dL (reference range: 0.1-0.2 mg/dL), alkaline phosphatase of 122 U/L (reference range: 25-125 U/L), aspartate transferase (AST) of 84 U/L (reference range: 5-40 U/L), alanine transaminase (ALT) of 36 U/L (5-50 U/L), international normalized ratio (INR) of 1.69 (<5.0), fibrinogen less than 50 mg/dL (reference range: 200-400 mg/dL), ferritin greater than 7,500 ng/mL (reference range: 11-310 ng/mL), factor VIII activity assay of 206% (reference range: 50-200%), ammonia level elevated at 227 U/L (reference range: 10-47 U/L). The patient also had a recent sickle hemoglobin (HbS) of 21.4% (reference range: 0%). A CT scan of the abdomen showed a distended gallbladder with cholelithiasis and hepatomegaly and increased density consistent with secondary hemochromatosis (Figures 1-2).

**Figure 3:**
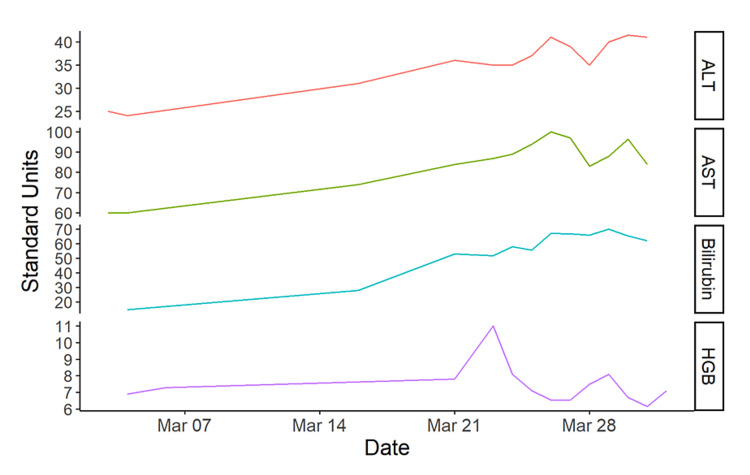
Graphical trend of hepatic function tests prior to and during hospitalization aspartame transferase (AST) and alanine transaminase (ALT) units: U/L; bilirubin units: mg/dL; hemoglobin (Hgb) units: g/dL.

**Figure 4:**
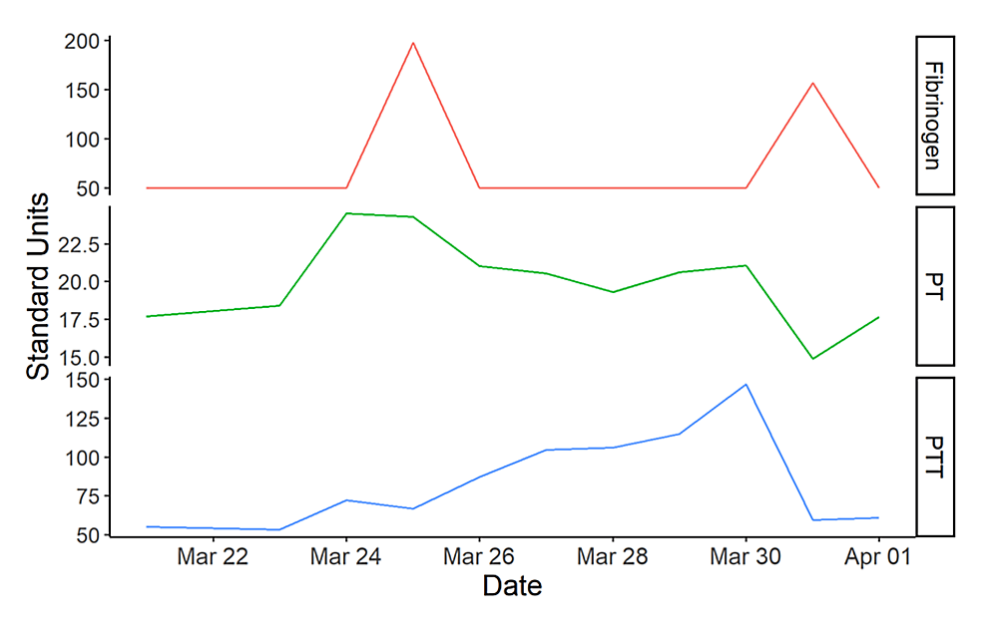
Graphical trend of coagulation studies prior to and during hospitalization fibrinogen units: mg/dL; prothrombin time (PT) units: seconds; partial thromboplastin time (PTT) units: seconds

The patient was admitted to the floor and given cryoprecipitate and packed red blood cells for supportive care. Hematology and biliary gastroenterology were consulted for worsening hyperbilirubinemia and a history of SCD. An abdominal ultrasound was obtained, which re-characterized a distended gallbladder with stones present without any intrahepatic or extrahepatic biliary dilatation. A hepatobiliary iminodiacetic acid (HIDA) scan was performed to rule out acute cholecystitis but was non-diagnostic due to decreased hepatic uptake of the tracer. Due to HbS being close to 20%, no exchange transfusion was performed on admission. During hospitalization, the patient continued to require packed red blood cells, cryoprecipitate, and vitamin K for a worsening blood count and INR (Figures 3-4).

Her hospital course was also complicated by worsening bleeding at her dialysis access site, oral mucosa, and bright red blood per rectum, despite not administering any heparin during dialysis. A colonoscopy was performed, which showed diffuse blood and clots from the cecum to the rectum without a clear source of bleeding. The patient’s mental status also began to wax and wane throughout the hospitalization, and she started to not tolerate her hemodialysis sessions. She was later transferred to the ICU after becoming hypothermic and hypotensive. She was started on a norepinephrine and vasopressin infusion and intubated for airway protection. She was also started on broad-spectrum IV antibiotics and stress-dose steroids without improvement. Palliative care was consulted during admission, and after discussions with the patient’s family, she was transitioned to comfort care. She was compassionately extubated and passed later that day.

## Discussion

Late presentations of liver failure were previously rare in clinical practice, given the high mortality of SCD. With advancements in therapeutics for SCD, patients with SCD have increased lifespans, and with them, patients are placed at increased risk for chronic organ failures. In our patient, she had several chronic organ failures, including ESRD requiring long-term intermittent hemodialysis and chronic liver disease from acquired hemochromatosis. In addition to chronic disease, patients with SCD can have acute exacerbations of their chronic problems. There are a few known acute sickle cell hepatopathies that are present in a spectrum of diseases, including acute sickle cell hepatic crisis, acute hepatic sequestration, and acute intrahepatic cholestasis [5-6].

The most severe of the acute hepatic complications that can occur with SCD is intrahepatic cholestasis. Intrahepatic cholestasis is characterized by signs of acute inflammation in the liver, including leukocytosis, fever, right upper quadrant (RUQ) abdominal pain, and jaundice [5]. On laboratory markers, the total serum bilirubin is typically significantly elevated, up to extreme levels. This is due to the acute sickling of erythrocytes within the hepatic sinusoids [7]. This in turn can cause hypoxia, resulting in the ballooning of hepatocytes and intracanalicular cholestasis [7]. In severe cases, acute sickling can cause local ischemia within the liver, leading to areas of hepatic necrosis on histology review [5]. Typically, the HbS concentration should be less than 20%-30% to help reduce the risk of sickling [8]. This can be acutely obtained by performing exchange transfusions with packed red blood cells. This was not performed on our patient, given that her recent HbS was borderline normal. If exchange transfusions are not possible or feasible, treatment is focused on supportive care with aggressive correction of coagulopathies [7].

Unfortunately, due to the patient’s transfusion dependence, she also had chronic liver disease due to iron overload. Before admission, she was treated with iron chelation but had recently stopped taking it due to ESRD and GI intolerance. Iron overload and acquired hemochromatosis are known complications of long-term transfusion dependence, including mortality, and should be discussed with any patient undergoing multiple red blood cell transfusions [9]. Our patient had significantly elevated ferritin levels as well as hepatic changes on a CT scan concerning iron deposition. This could be confirmed on a liver biopsy, but given our patient’s coagulopathy, a biopsy was not pursued [9]. Eventually, the liver can develop chronic cirrhosis from the acquired hemochromatosis, and the patient is at risk for acute decompensation or ACLF [10].

Acute-on-chronic liver failure is a newer designation for patients who present with decompensated cirrhosis and have additional systemic inflammation and extrahepatic organ failure. Unlike decompensated cirrhosis, ACLF is usually triggered by an acute systemic inflammatory event, such as infection, viral infection, or alcoholic hepatitis [10]. For our patient, her triggering event was likely her acute intrahepatic cholestasis [11]. With ACLF, multiple organ failures can be seen because of the systemic nature of the disease. In fact, short-term mortality is estimated based on the severity of the multi-organ failure with the Chronic Liver Failure (CLIF)-Sequenced Organ Failure Assessment (SOFA) score [12]. The score considers hepatic function as well as coagulation, renal function, respiratory status, neurological status, and hemodynamic status. Treatment for ACLF is centered around treatment of the precipitating event as well as supportive care in the ICU if needed [10]. For candidates, liver transplantation can be a viable treatment option if there is an accepting transplant center available [10].

## Conclusions

There are very few cases reported of ACLF from intrahepatic cholestasis in the sickle cell population. The mainstay of treatment for intrahepatic cholestasis is exchange transfusion, which should be initiated as soon as possible when the patient is identified as an appropriate candidate. If there is a progression of intrahepatic cholestasis to acute liver failure, or ACLF, supportive care and referral to a liver transplant center are appropriate next steps.
